# Effects of long-term therapy with quetiapine and olanzapine on parameters of immunity and cytokine levels in patients with schizophrenia

**DOI:** 10.1192/j.eurpsy.2021.2025

**Published:** 2021-08-13

**Authors:** O. Lobacheva, V. Nikitina, E. Kornetova, S. Vladimirova, A. Semke

**Affiliations:** 1 Laboratory Of Clinical Psychoneuroimmunology And Neurobiology, Tomsk National Research Medical Center, Tomsk, Russian Federation; 2 Department Of Endogenous Disorders, Mental Health Research Institute, Tomsk National Research Medical Center, Russian Academy of Sciences, Tomsk, Russian Federation; 3 Department Of Coordination Of Research, Mental Health Research Institute, Tomsk National Research Medical Center, Russian Academy of Sciences, Tomsk, Russian Federation

**Keywords:** Psychoneuroimmunology, schizophrénia, Antipsychotics

## Abstract

**Introduction:**

The study of effects of long-term antipsychotic therapy in patients with schizophrenia is relevant.

**Objectives:**

To study effects of long-term antipsychotic therapy on parameters of immunity and cytokine levels in patients with schizophrenia.

**Methods:**

We examined 20 schizophrenic patients, who received quetiapine (group 1) and 17 - olanzapine (group 2) for more than 6 months before admission in the hospital as the main anti-recurrence therapy. Persons aged 20-63 years with length of the follow-up of the disease ≥1 year were included. The investigations included: phenotyping of immunocompetent cells into CD differentiation clusters by flow cytometry; mitogen-induced, spontaneous production of cytokines (IL2, IFN-γ, IL-4, TNF-α) were identified with use of kits for enzyme-linked immunosorbent assay (ELISA).

**Results:**

It was shown that patients of group 1 in comparison with group 2 were characterized by lower values of СD3- lymphocytes (p=0.049), higher values of the spontaneous production of IFN-γ (p=0.01), mitogen-induced production of IL-2 (p=0.043) and IL-4 (p=0.059). In all examined low level of mitogen-induced of IFN-γ (p=0.0001) and TNFα (p=0.002; p=0.0001), high level of spontaneous production of TNFα (p=0.001) were revealed in relation to control.

**Conclusions:**

It was found that the acute period of schizophrenia after prolonged treatment with atypical antipsychotics is accompanied by immunological imbalance and dysregulation of the cytokine system. More severe immune disorders when hospitalized during the exacerbation period were revealed in patients who had been receiving antipsychotic therapy with the atypical antipsychotic quetiapine for a long time. This can be associated with the features of the mechanism of action of atypical antipsychotics.

**Disclosure:**

No significant relationships.

